# Three-Dimensional Models in Hepatic Surgery: Clinical Outcomes A Single-Center Experience

**DOI:** 10.3390/jcm14248659

**Published:** 2025-12-06

**Authors:** María Victoria Vieiro Medina, Laura Alonso Murillo, Carlos Ernesto García Vasquez, Marta de la Fuente Bartolomé, Victor Nieto Barros, Fernando Neria, Santos Jiménez de los Galanes Marchán

**Affiliations:** 1 General and Digestive Surgery Department, Infanta Elena University Hospital, 28342 Valdemoro-Madrid, Spain; laura.alonsom@quironsalud.es (L.A.M.); cgarciava@quironsalud.es (C.E.G.V.); santos.jimenez@quironsalud.es (S.J.d.l.G.M.); 2 Faculty of Medicine, Francisco de Vitoria University, 28223 Madrid, Spain; fernando.neria@ufv.es

**Keywords:** imaging, printing, three-dimensional, hepatic, surgery

## Abstract

**Background:** Hepatic resection requires precise knowledge of vascular anatomy and remnant liver volume to guarantee both safety and efficacy. Three-dimensional (3D) models, either virtual or printed, have been proposed as tools to optimize surgical planning, education, and intraoperative navigation. **Material and Methods:** This retrospective observational study evaluated the impact of 3D model utilization (virtual and printed), in 89 patients who underwent elective hepatectomy at Infanta Elena University Hospital (Valdemoro, Madrid, Spain) between May 2018 and May 2023. The implementation of 3D modeling began to be routinely implemented as of November 2020. Patients were divided into two groups: those without 3D modeling (*n* = 40) and those with 3D modeling (*n* = 49). **Results:** Baseline characteristics were comparable between groups. Intraoperative blood loss was significantly lower in the 3D model group (median 175 mL vs. 262.5 mL; *p* < 0.001), with no statistically significant differences in operative time, complication rate (Clavien–Dindo classification), length of hospital stay, or in-hospital mortality. Multivariable analysis identified dyslipidemia, postoperative sodium delta, and postoperative increase in direct bilirubin as independent risk factors for complications, whereas albumin demonstrated a protective effect. **Conclusions:** Three-dimensional modeling improves anatomic orientation and reduces intraoperative blood loss, although it does not significantly modify classic perioperative outcomes. Its principal value appears to reside in preoperative planning and technical safety rather than direct clinical impact.

## 1. Introduction

The liver possesses a highly complex vascular system, making it essential for surgeons to accurately understand the spatial relationships between hepatic vessels and tumor location to ensure safe and effective resections. Moreover, precise estimation of the future liver remnant is particularly important in patients with impaired hepatic function to prevent postoperative complications [1].

While conventional two-dimensional (2D) imaging methods such as computed tomography (CT) and magnetic resonance imaging (MRI) provide approximate information on hepatic anatomy, three-dimensional (3D) reconstruction enables the creation of individualized virtual or printed models that depict hepatic structures and spatial relationships in detail. These models offer a multi-angle visualization of intrahepatic vasculature, anatomic variations, lesion extent, and spatial measurements relative to adjacent vessels [2].

Three-dimensional simulation software has become an effective tool for visualizing vascular anatomy and estimating liver volume, and its application has been reported for anatomical analysis, volumetric assessment, vascular territory evaluation, and surgical planning [3]. Both virtual and printed 3D models have demonstrated benefits in preoperative planning, education, and intraoperative orientation. Printed models allow the hands-on visualization of patient-specific anatomy, assisting surgical decision-making in complex cases requiring vascular reconstruction, and improving anatomical understanding for both surgeons and trainees [4,5,6,7,8,9,10].

Among the reported advantages are improved surgical planning precision, safer identification of critical structures, and enhanced opportunities for surgical simulation and training. Virtual models allow dynamic manipulation and resection simulation, whereas printed models serve as tangible intraoperative references [6,8,11,12,13,14].

Nevertheless, their widespread clinical implementation is limited by production cost and time. Furthermore, while these models enhance planning and educational value, their direct effect on postoperative outcomes remains uncertain, and intraoperative ultrasound continues to play a key role in lesion detection [5,6,7,9,10,13]. Overall, both printed and virtual 3D models are valuable tools for complex hepatic surgery, though further large-scale studies are needed to determine their clinical impact [5,10,15].

Three-dimensional model implementation may improve perioperative parameters such as vascular structure identification, delineation of the transection plane, and intraoperative orientation, thereby enhancing surgical safety [6,11,12,16]. However, no consistent evidence supports reductions in operative time or intraoperative blood loss, although subjective improvements in team confidence and perceived safety have been reported [5,6]. These models may also optimize resection planning, preserve vascular structures, and improve estimation of the future liver remnant, potentially contributing to better postoperative recovery [11,12,16].

Recent studies have highlighted their utility in reducing surgeon stress and improving vascular identification, while confirming that their primary benefits lie in educational and planning domains rather than direct clinical outcomes [1,5,6]. The multicenter LIV3DPRINT study validated the accuracy of 3D-printed models derived from imaging data for surgical planning and education, confirming good correlation with CT/MRI and pathological findings [5]. Similarly, Joo et al. demonstrated that personalized 3D-printed hepatic models improved lesion-by-lesion pathological correlation and staging accuracy for small focal liver lesions [17].

Over the past decade, 3D diagnostic models have gained increasing acceptance in hepatobiliary surgery, providing improved anatomical understanding and facilitating surgical planning. However, their true clinical impact on perioperative outcomes remains to be fully elucidated [18,19].

Based on these considerations, the present study aims to evaluate the effect of 3D model utilization in hepatic surgery with respect to perioperative outcomes.

## 2. Materials and Methods

A retrospective observational case-control study was conducted at Infanta Elena University Hospital (Valdemoro, Madrid, Spain). The primary objective was to determine the association between the use of 3D models (virtual and printed) and perioperative outcomes. The implementation of 3D modeling has been routinely implemented as of November 2020.

All patients who underwent hepatic resection with available preoperative imaging (CT or MRI) between May 2018 and May 2023 were included. Patients undergoing urgent procedures or simultaneous resections of other organs were excluded.

Patients were classified as cases if a preoperative 3D model had been generated, and as controls if no such model was used.

### 2.1. Variables and Data Collection

Variables were grouped into three categories:

*Demographic and Preoperative Variables:*

Age (years), sex, body mass index (BMI), relevant medical history, presence of steatosis on preoperative ultrasound, degree of fibrosis measured by preoperative FibroScan, number and location of lesions, and preoperative laboratory parameters, including hemoglobin, platelet count, international normalized ratio (INR), creatinine, total and direct bilirubin, aspartate aminotransferase (AST), alanine aminotransferase (ALT), alkaline phosphatase (ALP), gamma-glutamyl transferase (GGT), lactate dehydrogenase (LDH), sodium, and albumin. For malignant disease, tumor markers were recorded as follows: alpha-fetoprotein (AFP), carbohydrate antigen 19-9 (CA19-9), and carcinoembryonic antigen (CEA). Functional status was assessed by the Eastern Cooperative Oncology Group (ECOG) [20], the American Society of Anesthesiologists (ASA) physical status classification [21], and the Child–Pugh score [22]. Use of preoperative chemotherapy was also recorded.

*Intraoperative Variables:*

Type of surgical procedure performed, surgical approach (laparoscopic, open, or converted), operative time, and intraoperative blood loss.

*Postoperative Variables:*

Histopathologic diagnosis, percentage of steatosis in the resected specimen, postoperative complications classified according to the Clavien–Dindo classification [23], need for percutaneous drainage, administration of parenteral nutrition or antibiotics, reintervention rate, and in-hospital mortality. Length of stay in the intensive care unit (ICU) and total hospitalization were recorded. Delta values for key laboratory parameters were calculated as the difference between preoperative values and the last value obtained within the first 72 postoperative hours (hemoglobin, platelets, INR, total bilirubin, direct bilirubin, AST, ALT, ALP, GGT, LDH, sodium, and albumin).

### 2.2. Acquisition of 3D Models

**Figure 1:**
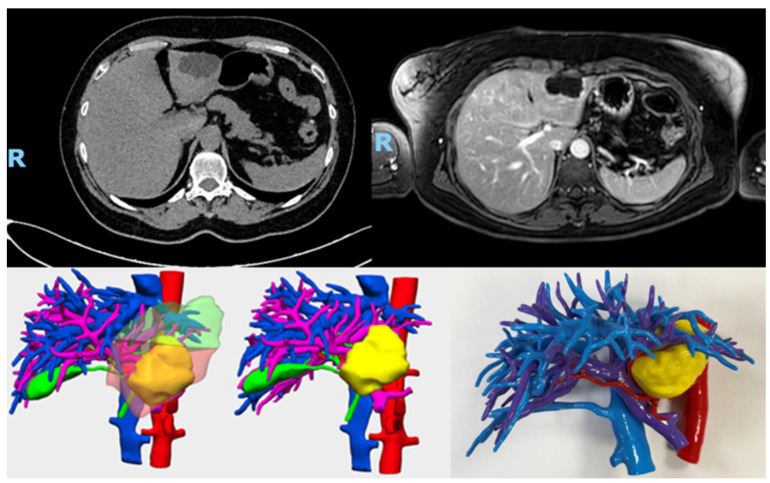
Virtual and printed 3D model overlaid on the 2D imaging (CT and MRI) of a cystic lesion in segments 2–3.

In the case group, each model was generated from high-resolution CT and/or MRI datasets using 3D-MSP software (Cella Medical Solutions, Murcia, Spain). Both virtual and printed models were used during preoperative planning, and the printed models were sterilized to allow their use as intraoperative three-dimensional anatomical references. Figure 1 illustrates an example of a virtual and printed 3D model overlaid on the 2D imaging (CT and MRI) of a cystic lesion in segments 2–3.

### 2.3. Surgical Technique

All hepatic resections were performed using an ultrasonic surgical aspirator (CUSA) and Aquamantys system. Intraoperative ultrasound was routinely performed in all cases. The type of resection was determined by tumor type, number, and extension.

### 2.4. Statistical Analysis

Continuous variables were expressed as median (interquartile range) and were compared using the Mann–Whitney U test (Wilcoxon rank-sum test).

Categorical variables were presented as absolute and relative frequencies and were compared using the Chi-squared test or Fisher’s exact test, as appropriate.

Boxplots were used to visualize variability in intraoperative blood loss between groups.

Variables with a *p*-value < 0.2 in univariate analysis, as well as the variable “3D model,” were included in a multivariable logistic regression model. Backward stepwise selection using the Akaike Information Criterion (AIC) was applied to obtain the final model.

A *p*-value < 0.05 was considered statistically significant. Statistical analysis was performed using R software, version 4.4.

## 3. Results

**Figure 2:**
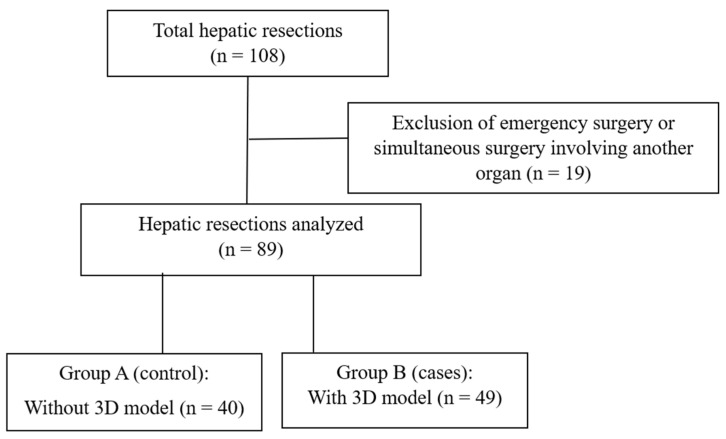
Study flow diagram. Of the 108 consecutive patients who underwent hepatic resections, 19 were excluded because they had undergone simultaneous surgery of another organ or emergency procedures. The remaining 89 patients were divided into two groups: those without preoperative 3D simulation (Group A, no 3D model) and those with preoperative 3D simulation (Group B, 3D model).

Between May 2018 and May 2023, a total of 108 patients underwent hepatic resection at Infanta Elena University Hospital (Valdemoro, Madrid, Spain). Nineteen patients were excluded for not meeting inclusion criteria (urgent procedures or simultaneous resection of another organ), leaving 89 patients for analysis. These were divided into two groups: those without preoperative 3D simulation (Group A, *n* = 40) and those with 3D simulation (Group B, *n* = 49) (Figure 2).

### 3.1. Baseline Characteristics

Demographic and preoperative characteristics of the 89 patients are summarized in Table 1. No significant differences were observed between groups, except for preoperative direct bilirubin levels, which were higher in the 3D group (*p* = 0.025). All patients were classified as Child–Pugh class A. Most patients had ECOG performance status 0 (*n* = 85), with only four patients classified as ECOG 1. ASA class II was the most frequent anesthetic risk category. A similar proportion of patients in both groups had received neoadjuvant chemotherapy (61.3% in Group A vs. 66.7% in Group B; *p* = 0.654).

### 3.2. Surgical Procedure and Intraoperative Findings

Details regarding the type of resection, surgical approach, operative time, and histologic steatosis are summarized in Table 2. The most frequent type of resection was single limited resection (32.5% in Group A vs. 24.5% in Group B). A laparoscopic approach was predominant in both groups (52.5% vs. 67.3%), while open surgery was performed in 47.5% and 30.6% of cases, respectively. Only one case of conversion from laparoscopy to open surgery occurred (2.0%, Group B). This patient presented with multiple hepatic metastases from a gastrointestinal stromal tumor (GIST), including a lesion located in segment 8, in close proximity to the inferior vena cava. Due to the high risk of vascular injury, conversion to open surgery was performed to ensure safety and adequate vascular control.

Operative time did not differ significantly between groups (median 226.5 [181.5–260.0] minutes in Group A vs. 225.0 [186.0–252.0] minutes in Group B; *p* = 0.528). Histopathological assessment of steatosis in resected specimens revealed no significant differences between groups, with most patients having <5% steatosis (50.0% vs. 65.8%).

### 3.3. Intraoperative Blood Loss

**Figure 3:**
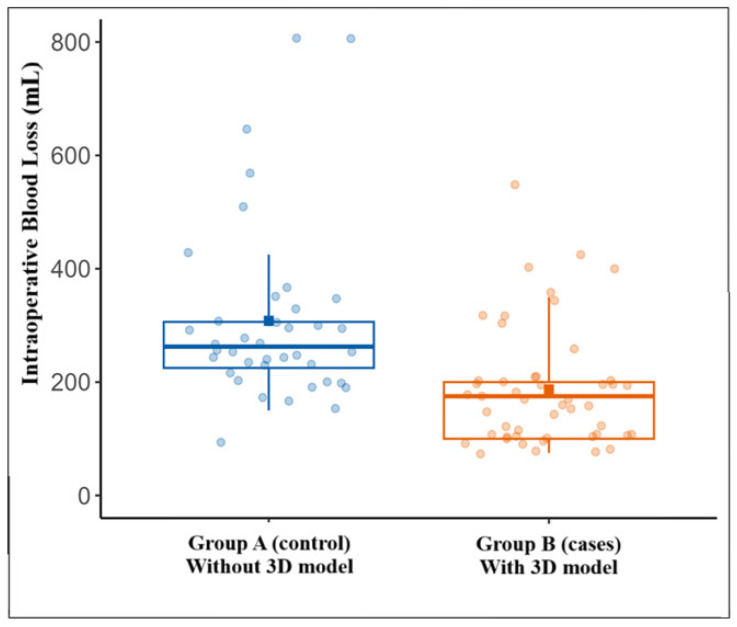
Intraoperative Blood Loss: Boxplot illustrating intraoperative blood loss, which differed significantly between the two groups (*p* < 0.001), with a median of 262.5 mL in Group A and 175.0 mL in Group B.

Intraoperative blood loss was significantly lower in the 3D model group (median 175 mL [100–200] vs. 262.5 mL [225–312.5] in controls; *p* < 0.001). This finding is depicted in the boxplot shown in Figure 3.

### 3.4. Postoperative Outcomes

Postoperative outcomes, including ICU and hospital length of stay, Clavien–Dindo complications, in-hospital mortality, need for antibiotics, total parenteral nutrition, percutaneous drainage, and reoperation rates, are summarized in Table 3. No statistically significant differences were observed between groups.

Median hospital stay was similar in both groups (6.0 [4.0–8.0] days in controls vs. 5.0 [4.0–7.0] days in the 3D group; *p* = 0.192). ICU admission rates were comparable (85.0% vs. 83.7%), as was ICU length of stay (median 1 day in both groups).

### 3.5. Laboratory Parameter Changes (Delta Values)

Delta values for key laboratory parameters are shown in Table 4. A statistically significant difference was observed only for delta INR, which was lower in the 3D group (median 0.08 [0.02–0.16] vs. 0.14 [0.09–0.22]; *p* = 0.01). No significant differences were found for other biochemical deltas (hemoglobin, platelets, bilirubin, sodium, albumin, or liver enzymes).

### 3.6. Multivariable Analysis

Variables associated with postoperative complications, according to the Clavien–Dindo classification, were analyzed both with and without consideration of 3D model use. Variables with *p* < 0.2 in univariate analysis, along with 3D model use, were included in the multivariable logistic regression. The final model, summarized in Table 5, identified preoperative GGT, BMI, age, the presence or absence of dyslipidemia, and changes (deltas) in direct bilirubin, sodium, and albumin as the most influential variables. These variables demonstrated the greatest statistical explanatory and predictive power for postoperative complications.

## 4. Discussion

In this series of 89 patients undergoing elective hepatic resection, the use of three-dimensional (3D) models, both virtual and printed, was found to be associated with a significant reduction in intraoperative blood loss (median 175 mL vs. 262.5 mL; *p* < 0.001) compared with the control group, without statistically significant differences in operative time, overall complication rate, hospital length of stay, or in-hospital mortality.

These results are consistent with the current literature, which highlights that the primary advantage of 3D technology lies in improved anatomical orientation and precise preoperative planning, facilitating recognition of the intrahepatic venous anatomy and definition of the transection plane. However, several studies report that these technical benefits do not consistently translate into reductions in operative time, blood loss, complications, or mortality, supporting the notion that the advantages are primarily technical rather than clinical [6].

Some investigators have reported that 3D planning may decrease intraoperative bleeding through more accurate transection line delineation and early identification of vascular variants, though this benefit is not consistently accompanied by shorter operative time or reduced postoperative complication rates [2,24,25]. Other studies have found no significant differences in intraoperative blood loss associated with 3D model use [1,5,6,26].

With respect to operative time, our findings revealed no significant difference between groups. A cross-sectional meta-analysis of solid-organ resections, including hepatic subgroups, demonstrated that 3D virtual planning was associated with a shorter overall operative time [1,25].

The lack of impact on hospital stay and overall morbidity is consistent with the findings of Cheng et al. (2022) [27] who emphasized that intraoperative technical improvements conferred by 3D models do not necessarily result in clinically meaningful short-term outcomes. Postoperative recovery is influenced by multiple factors, including baseline patient condition, comorbidities, extent of resection, anesthetic management, and perioperative care, which may outweigh the impact of preoperative simulation.

Our multivariable analysis showed that the strongest predictors of postoperative complications were patient-related factors—age, dyslipidemia, and early postoperative biochemical changes (delta sodium, albumin, and direct bilirubin)—rather than the use of 3D modeling. This underscores that metabolic profile and baseline physiology remain key determinants of postoperative risk, and that, while 3D technology offers technical advantages, it does not replace comprehensive preoperative assessment.

Dyslipidemia was identified as an independent risk factor (OR 7.88), a finding partially supported by the literature. Meta-analyses on hepatectomy outcomes in patients with metabolic syndrome, a condition inherently associated with dyslipidemia, have demonstrated higher rates of postoperative bleeding and infectious complications, though not of hepatobiliary-specific complications, such as bile leak or posthepatectomy liver failure (PHLF). Importantly, when metabolic syndrome is associated with histologically confirmed non-alcoholic fatty liver disease (NAFLD), the risk of PHLF is significantly increased, though overall survival remains comparable to patients without the syndrome [28].

Postoperative sodium changes were also significantly associated with complications, in agreement with studies reporting that hyponatremia increases the risk of adverse postoperative events, particularly in cirrhotic patients, who are more susceptible to systemic complications due to underlying pathophysiology [29,30].

Serum albumin demonstrated a protective effect: a greater postoperative decline was associated with higher complication rates. This is consistent with existing evidence showing that both low preoperative albumin levels and significant early postoperative albumin decreases are robust predictors of postoperative complications and can be used for risk stratification and closer postoperative monitoring [31,32,33,34].

Similarly, postoperative bilirubin elevation is a well-recognized marker of hepatic dysfunction and is independently associated with major complications, regardless of resection extent [35,36].

Interestingly, BMI in our cohort showed an inverse association with postoperative complications. This finding contrasts with most reports in the literature [37,38,39], and may represent a statistical artifact or residual confounding, as BMI could be influenced by age, sarcopenia, frailty, or residual liver volume. In addition, sample distribution and the lack of BMI categorization may introduce selection bias, warranting cautious interpretation.

This study has several limitations. Its retrospective, single-center design, lack of randomization, and relatively small sample size inherently limit the generalizability of the findings. Although our subgroup analysis did not identify significant differences between major and minor resections, 3D modeling may provide greater benefit in complex or major hepatectomies, as suggested by previous literature [16]. Future research should include prospective, multicenter studies, ideally randomized controlled trials with larger sample sizes, to determine whether the systematic integration of 3D planning can lead to meaningful reductions in morbidity and improved long-term outcomes after hepatectomy.

## 5. Conclusions

This study demonstrates that the use of three-dimensional (3D) models, both virtual and printed, for preoperative planning in hepatic resection is associated with a significant reduction in intraoperative blood loss, but with no demonstrable impact on operative time, overall postoperative morbidity, hospital stay, or in-hospital mortality.

These findings reinforce the concept that the primary value of this technology lies in enhancing anatomical orientation and technical safety rather than directly improving classical clinical outcomes.

Multivariable analysis highlights that patient-related factors, including age, dyslipidemia, and early postoperative biochemical alterations (hyponatremia, hypoalbuminemia, and direct bilirubin elevation), are more closely associated with postoperative complications than 3D model utilization. This underscores the need for comprehensive, individualized preoperative assessment, with 3D simulation serving as a complement rather than a substitute for clinical risk stratification.

Although available evidence supports consistent technical benefits, the lack of clinically significant improvements in this study supports the need for larger, prospective, multicenter trials to establish the true impact of 3D modeling on perioperative and long-term outcomes in hepatic surgery.

## Figures and Tables

**Table 1:** Demographic and preoperative patient characteristics.

	3D Models		
	No	Yes	*p*-Value ^1^
	*n* = 40	*n* = 49	
Age, years			0.594
Mean ± SD	62.9 ± 10.2	60.6 ± 12.4	
Median [IQR]	62.0 [54.5–70.5]	61.0 [53.0–72.0]	
Sex, n (%)			0.154
Female	19 (47.5%)	16 (32.7%)	
Male	21 (52.5%)	33 (67.3%)	
BMI, kg/m^2^			0.843
Mean ± SD	28.00 ± 5.79	28.05 ± 4.42	
Median [IQR]	27.16 [23.70–32.53]	27.68 [25.30–29.74]	
Diabetes Mellitus, n (%)	9 (22.5%)	6 (12.2%)	0.199
Hypertension, n (%)	24 (60.0%)	21 (42.9%)	0.108
Dyslipidemia, n (%)	11 (27.5%)	6 (12.2%)	0.069
Arrhythmia/Valvular disease, n (%)	2 (5.0%)	2 (4.1%)	>0.999
Respiratory history, n (%)	8 (20.0%)	5 (10.2%)	0.193
Type, n (%)			>0.999
COPD	3 (42.9%)	2 (40.0%)	
OSA	1 (14.3%)	2 (40.0%)	
COPD + OSA	1 (14.3%)	0 (0.0%)	
Bronchial asthma	1 (14.3%)	0 (0.0%)	
Pulmonary fibrosis	0 (0.0%)	1 (20.0%)	
Pulmonary sarcoidosis	1 (14.3%)	0 (0.0%)	
Not available	33	44	
Steatosis on ultrasound, n (%)	10 (33.3%)	9 (26.5%)	0.549
Not available	10	15	
Fibroscan, n (%)			0.212
F0-1 (less than 8.2 kPa)	1 (33.3%)	1 (12.5%)	
F2 (8.2–9.7 kPa)	0 (0.0%)	4 (50.0%)	
F3 (9.7–13.6 kPa)	0 (0.0%)	2 (25.0%)	
F4 (13.6 kPa or higher)	2 (66.7%)	1 (12.5%)	
Not available	37	41	
Number of lesions			0.491
Mean ± SD	2.5 ± 4.8	1.9 ± 1.8	
Median [IQR]	1.0 [1.0–2.0]	1.0 [1.0–2.0]	
Lesions by Category, n (%)			0.427
Single	24 (60.0%)	35 (71.4%)	
2–4	13 (32.5%)	10 (20.4%)	
5 or more	3 (7.5%)	4 (8.2%)	
Location, n (%)			0.311
Unilobar	30 (75.0%)	41 (83.7%)	
Bilobar	10 (25.0%)	8 (16.3%)	
Hemoglobin (g/dL)			0.145
Mean ± SD	13.5 ± 1.6	13.9 ± 1.5	
Median [IQR]	13.3 [12.6–14.6]	13.9 [13.0–15.2]	
Platelet count (×10^3^/µL)			0.665
Mean ± SD	225.3 ± 85.0	213.3 ± 76.5	
Median [IQR]	210.5 [170.0–256.5]	210.0 [146.0–277.0]	
INR			0.795
Mean ± SD	1.06 ± 0.10	1.10 ± 0.21	
Median [IQR]	1.07 [1.00–1.11]	1.06 [1.01–1.12]	
Creatinine (mg/dL)			0.063
Mean ± SD	0.79 ± 0.19	0.86 ± 0.18	
Median [IQR]	0.79 [0.64–0.90]	0.84 [0.73–0.95]	
Total bilirubin (mg/dL)			0.079
Mean ± SD	0.55 ± 0.36	0.69 ± 0.42	
Median [IQR]	0.45 [0.34–0.60]	0.59 [0.40–0.90]	
Direct bilirubin (mg/dL)			0.025 *
Mean ± SD	0.18 ± 0.10	0.25 ± 0.19	
Median [IQR]	0.15 [0.10–0.22]	0.20 [0.15–0.28]	
AST (U/L)			0.308
Mean ± SD	26.6 ± 25.5	27.7 ± 17.1	
Median [IQR]	18.5 [16.0–29.5]	22.0 [17.0–30.0]	
ALT (U/L)			0.558
Mean ± SD	26.8 ± 25.2	30.1 ± 27.4	
Median [IQR]	21.0 [13.0–31.0]	20.0 [15.0–32.0]	
ALP (U/L)			0.66
Mean ± SD	115.8 ± 130.8	97.7 ± 44.3	
Median [IQR]	89.5 [76.0–114.0]	89.0 [67.0–113.0]	
GGT (U/L)			0.83
Mean ± SD	72.6 ± 98.3	60.7 ± 71.7	
Median [IQR]	35.0 [20.0–78.0]	31.5 [23.0–70.0]	
LDH (U/L)			0.809
Mean ± SD	209.9 ± 79.9	205.5 ± 71.3	
Median [IQR]	186.0 [165.0–230.0]	191.0 [152.0–239.0]	
Sodium (mmol/L)			0.686
Mean ± SD	140.0 ± 3.3	140.4 ± 2.5	
Median [IQR]	140.0 [138.0–142.0]	140.0 [139.0–142.0]	
Albumin (g/dL)			0.481
Mean ± SD	4.11 ± 0.41	4.19 ± 0.40	
Median [IQR]	4.15 [3.95–4.35]	4.20 [4.00–4.50]	
AFP (ng/mL)			0.591
Mean ± SD	4.30 ± 3.88	21.61 ± 57.10	
Median [IQR]	2.11 [1.70–8.87]	3.03 [2.20–5.29]	
Not available	33	39	
CA19.9 (U/mL)			0.939
Mean ± SD	313.5 ± 930.8	37.7 ± 83.5	
Median [IQR]	10.0 [5.0–80.0]	11.0 [8.0–14.0]	
Not available	29	40	
CEA (ng/mL)			0.566
Mean ± SD	21.3 ± 78.3	12.4 ± 41.1	
Median [IQR]	3.6 [1.8–5.1]	2.9 [1.9–4.4]	
Not available	12	30	
ECOG, n (%)			0.624
ECOG 0	39 (97.5%)	46 (93.9%)	
ECOG 1	1 (2.5%)	3 (6.1%)	
ASA, n (%)			0.203
ASA 1	3 (7.5%)	3 (6.1%)	
ASA 2	16 (40.0%)	29 (59.2%)	
ASA 3	21 (52.5%)	17 (34.7%)	
Child–Pugh classification, n (%)			0.849
5	35 (87.5%)	44 (89.8%)	
6	5 (12.5%)	4 (8.2%)	
7	0 (0.0%)	1 (2.0%)	
Preoperative chemotherapy, n (%)	19 (61.3%)	22 (66.7%)	0.654
Not available	9	16	

^1^ Wilcoxon rank sum test; Pearson’s Chi-squared test; Fisher’s exact test. * Statistically significant difference. Abbreviations; BMI: body mass index; COPD: chronic obstructive pulmonary disease; OSA: obstructive sleep apnea; INR: international normalized ratio; AST: aspartate aminotransferase; ALT: alanine aminotransferase; ALP: alkaline phosphatase; GGT: gamma-glutamyl transferase; LDH: lactate dehydrogenase; AFP: alpha-fetoprotein, CA19-9: carbohydrate antigen 19-9; CEA: carcinoembryonic antigen; ECOG: Eastern Cooperative Oncology Group (ECOG) performance status; ASA: American Society of Anesthesiologists physical status classification.

**Table 2:** Surgical procedure and intraoperative findings.

	3D Models		
	No	Yes	*p*-Value ^1^
	*n* = 40	*n* = 49	
Type of surgery, n (%)			0.512
Single limited resection	13 (32.5%)	12 (24.5%)	
Multiple limited resection	8 (20.0%)	11 (22.4%)	
Bisegmentectomy	7 (17.5%)	9 (18.4%)	
Hepatic cystectomy	3 (7.5%)	7 (14.3%)	
Segmentectomy	2 (5.0%)	5 (10.2%)	
Right hemihepatectomy	2 (5.0%)	2 (4.1%)	
Left hemihepatectomy	1 (2.5%)	3 (6.1%)	
Hepatic cysts fenestration	3 (7.5%)	0 (0.0%)	
Extended right hepatectomy	1 (2.5%)	0 (0.0%)	
Surgical approach, n (%)			0.127
Open	19 (47.5%)	15 (30.6%)	
Laparoscopic	21 (52.5%)	33 (67.3%)	
Converted laparoscopy	0 (0.0%)	1 (2.0%)	
Operative time (min)			0.528
Mean ± SD	232.5 ± 74.7	219.2 ± 48.1	
Median [IQR]	226.5 [181.5–260.0]	225.0 [186.0–252.0]	
Steatosis on histopathology, n (%)			0.334
None (<5%)	17 (50.0%)	25 (65.8%)	
Mild (5–33%)	13 (38.2%)	9 (23.7%)	
Moderate (34–66%)	4 (11.8%)	4 (10.5%)	
Severe (>66%)	0 (0.0%)	0 (0.0%)	
Not available	6	11	

^1^ Fisher’s exact test; Wilcoxon rank sum test; Pearson’s Chi-squared test.

**Table 3:** Postoperative outcomes.

	3D Models		
	No	Yes	*p*-Value ^1^
	*n* = 40	*n* = 49	
Clavien–Dindo n (%)			0.516
No complication	26 (65.0%)	35 (71.4%)	
With complication	14 (35.0%)	14 (28.6%)	
Clavien–Dindo classification, n (%)			
Grade I	2 (14.3%)	1 (7.1%)	
Grade II	11 (78.6%)	4 (28.6%)	
Grade IIIa	0 (0.0%)	3 (21.4%)	
Grade IIIb	0 (0.0%)	5 (35.7%)	
Grade Iva	0 (0.0%)	1 (7.1%)	
Grade V	1 (7.1%)	0 (0.0%)	
Not available (N/A)	26	35	
Postoperative percutaneous drainage, n (%)	0 (0.0%)	4 (8.2%)	0.124
Parenteral nutrition, n (%)	4 (10.0%)	4 (8.2%)	>0.999
Antibiotics, n (%)	5 (12.5%)	6 (12.2%)	>0.999
Reoperation, n (%)	0 (0.0%)	1 (2.0%)	>0.999
In-hospital mortality, n (%)	1 (2.5%)	0 (0.0%)	0.449
Hospital stay			0.192
Mean ± SD	7.4 ± 5.5	7.0 ± 7.0	
Median [IQR]	6.0 [4.0–8.0]	5.0 [4.0–7.0]	
Intensive care unit stay, n (%)	34 (85.0%)	41 (83.7%)	0.864
Intensive care unit stay (days)			0.116
Mean ± SD	1.9 ± 1.7	1.8 ± 2.0	
Median [IQR]	1.0 [1.0–2.0]	1.0 [1.0–2.0]	
Not available	6	8	

^1^ Pearson’s Chi-squared test; Fisher’s exact test; Wilcoxon rank sum test.

**Table 4:** Laboratory parameter changes (delta values).

	3D Models		
	No	Yes	*p*-Value ^1^
	*n* = 40	*n* = 49	
Delta hemoglobin			0.735
Mean ± SD	−2.43 ± 1.41	−2.46 ± 1.44	
Median [IQR]	−2.35 [−3.35–−1.45]	−2.60 [−3.40–−1.50]	
Delta platelet count			0.85
Mean ± SD	−55.4 ± 82.3	−49.2 ± 63.4	
Median [IQR]	−51.0 [−77.0–−28.5]	−47.0 [−84.0–−23.0]	
Delta INR			0.01 *
Mean ± SD	0.19 ± 0.35	0.08 ± 0.21	
Median [IQR]	0.14 [0.09–0.22]	0.08 [0.02–0.16]	
Delta total bilirubin			0.433
Mean ± SD	0.09 ± 0.39	0.00 ± 0.47	
Median [IQR]	0.08 [−0.10–0.31]	0.05 [−0.20–0.25]	
Delta direct bilirubin			0.362
Mean ± SD	0.14 ± 0.23	0.07 ± 0.18	
Median [IQR]	0.07 [0.03–0.13]	0.06 [−0.03–0.15]	
Not available	2	2	
Delta GOT			0.944
Mean ± SD	347.9 ± 1389.8	104.3 ± 114.4	
Median [IQR]	68.0 [13.5–158.5]	63.0 [20.0–164.0]	
Delta GPT			0.993
Mean ± SD	331.43 ± 516.71	256.94 ± 261.16	
Median [IQR]	162.00 [91.00–349.00]	162.00 [81.00–391.00]	
Delta ALP			0.436
Mean ± SD	−6.0 ± 29.9	−10.7 ± 42.5	
Median [IQR]	−4.0 [−21.0–6.0]	−11.0 [−25.0–10.0]	
Not available	2	0	
Delta GGT			0.383
Mean ± SD	14.0 ± 60.1	8.1 ± 55.2	
Median [IQR]	8.0 [−4.0–41.0]	6.0 [−12.0–22.0]	
Not available	11	6	
Delta LDH			0.056
Mean ± SD	14.4 ± 154.2	46.5 ± 74.7	
Median [IQR]	15.5 [−53.0–53.0]	37.0 [5.5–74.5]	
Not available	2	1	
Delta Sodium			0.958
Mean ± SD	−0.61 ± 3.56	−0.60 ± 3.27	
Median [IQR]	−1.00 [−3.00–2.00]	0.00 [−3.00–2.00]	
Not available	2	2	
Delta albumin			0.195
Mean ± SD	−0.98 ± 0.49	−0.86 ± 0.45	
Median [IQR]	−1.10 [−1.35–−0.75]	−0.90 [−1.20–−0.60]	
Not available	0	2	

^1^ Wilcoxon rank sum test. * Statistically significant difference. Abbreviations; BMI: body mass index; COPD: chronic obstructive pulmonary disease; OSA: obstructive sleep apnea; INR: international normalized ratio; AST: aspartate aminotransferase; ALT: alanine aminotransferase; ALP: alkaline phosphatase; GGT: gamma-glutamyl transferase; LDH: lactate dehydrogenase.

**Table 5:** Multivariate model adjusted using a backward stepwise algorithm based on the Akaike Information Criterion (AIC).

	OR	95% CI	*p*-Value
GGT	1.01	1.00, 1.03	0.013 *
BMI	0.89	0.74, 1.02	0.123
Age	1.07	1.01, 1.16	0.047 *
Delta direct bilirubin	26,8	1.19, 1.369	0.054
Dyslipidemia			
Yes	7.88	1.46, 54.5	0.023 *
Delta sodium	1.25	1.02, 1.60	0.048 *
Delta albumin	0.18	0.02, 0.93	0.063
3D Model			
Yes	1.64	0.41, 7.16	0.493

* Statistically significant difference. Abbreviations: CI: confidence interval, OR: odds ratio, GGT: gamma-glutamyl transferase; BMI: body mass index.

## Data Availability

Datasets analyzed during the current study are not publicly available due to patient privacy limitations, but are available from the corresponding author on reasonable request.

## Abbreviations

The following abbreviations are used in this manuscript:

2D	Two-dimensional
CT	Computed tomography
MRI	Magnetic resonance
3D	Three-dimensional
BMI	Body mass index
INR	International normalized ratio
AST	Aspartate aminotransferas
ALT	Alanine aminotransferase
ALP	Alkaline phosphatase
GGT	Gamma-glutamyl transferase
LDH	Lactate dehydrogenase
AFP	Alpha-fetoprotein
CA 19,9	Carbohydrate antigen 19-9
CEA	Carcinoembryonic antigen
ECOG	Eastern Cooperative Oncology Group
ASA	American Society of Anesthesiologists
ICU	Intensive care unit
CUSA	Ultrasonic surgical aspirator
AIC	Akaike Information Criterion
PHLF	Posthepatectomy liver failure
NAFLD	Non-alcoholic fatty liver disease.
